# *CTLA4* mRNA is downregulated by miR-155 in regulatory T cells, and reduced blood *CTLA4* levels are associated with poor prognosis in metastatic melanoma patients

**DOI:** 10.3389/fimmu.2023.1173035

**Published:** 2023-05-01

**Authors:** Prasanna Kumar Vaddi, Douglas Grant Osborne, Andrew Nicklawsky, Nazanin K. Williams, Dinoop Ravindran Menon, Derek Smith, Jonathan Mayer, Anna Reid, Joanne Domenico, Giang Huong Nguyen, William A. Robinson, Melanie Ziman, Dexiang Gao, Zili Zhai, Mayumi Fujita

**Affiliations:** ^1^ Department of Dermatology, University of Colorado Anschutz Medical Campus, Aurora, CO, United States; ^2^ University of Colorado Cancer Center Biostatistics Core, University of Colorado Anschutz Medical Campus, Aurora, CO, United States; ^3^ Department of Medicine, University of Colorado Anschutz Medical Campus, Aurora, CO, United States; ^4^ School of Medical and Health Sciences, Edith Cowan University, Perth, WA, Australia; ^5^ School of Biomedical Science, University of Western Australia, Perth, WA, Australia; ^6^ Department of Immunology, University of Colorado Anschutz Medical Campus, Aurora, CO, United States; ^7^ Department of Veterans Affairs Medical Center, VA Eastern Colorado Health Care System, Aurora, CO, United States

**Keywords:** melanoma, CTLA-4, biomarker, regulatory T cells, miRNA-155

## Abstract

Cytotoxic T lymphocyte-associated antigen-4 (CTLA-4) is an immune checkpoint expressed in regulatory T (Treg) cells and activated T lymphocytes. Despite its potential as a treatment strategy for melanoma, CTLA-4 inhibition has limited efficacy. Using data from The Cancer Genome Atlas (TCGA) melanoma database and another dataset, we found that decreased *CTLA4* mRNA was associated with a poorer prognosis in metastatic melanoma. To investigate further, we measured blood *CTLA4* mRNA in 273 whole-blood samples from an Australian cohort and found that it was lower in metastatic melanoma than in healthy controls and associated with worse patient survival. We confirmed these findings using Cox proportional hazards model analysis and another cohort from the US. Fractionated blood analysis revealed that Treg cells were responsible for the downregulated *CTLA4* in metastatic melanoma patients, which was confirmed by further analysis of published data showing downregulated CTLA-4 surface protein expression in Treg cells of metastatic melanoma compared to healthy donors. Mechanistically, we found that secretomes from human metastatic melanoma cells downregulate *CTLA4* mRNA at the post-transcriptional level through miR-155 while upregulating *FOXP3* expression in human Treg cells. Functionally, we demonstrated that *CTLA4* expression inhibits the proliferation and suppressive function of human Treg cells. Finally, miR-155 was found to be upregulated in Treg cells from metastatic melanoma patients compared to healthy donors. Our study provides new insights into the underlying mechanisms of reduced *CTLA4* expression observed in melanoma patients, demonstrating that post-transcriptional silencing of *CTLA4* by miRNA-155 in Treg cells may play a critical role. Since CTLA-4 expression is downregulated in non-responder melanoma patients to anti-PD-1 immunotherapy, targeting miRNA-155 or other factors involved in regulating *CTLA4* expression in Treg cells without affecting T cells could be a potential strategy to improve the efficacy of immunotherapy in melanoma. Further research is needed to understand the molecular mechanisms regulating *CTLA4* expression in Treg cells and identify potential therapeutic targets for enhancing immune-based therapies.

## 1 Introduction

Melanoma is one of the aggressive forms of skin cancer. Its incidence increases by over 3% annually and continues to rise in Caucasians (1). Until recently, the survival rate for advanced melanoma patients was around 10% (2). However, recent advances in our understanding of tumor immunology and cancer biology have led to promising developments of immune checkpoint inhibitors and targeted therapies, resulting in significant improvements in long-term survival, with a substantial subset of melanoma patients experiencing durable responses (3). Despite these advances, some patients do not respond to immunotherapy or experience limited benefits, and approximately two-thirds of patients treated with immune checkpoint monotherapies eventually progress (4), underscoring the importance of understanding the mechanisms of immune evasion, drug resistance, and poor prognosis in these cancer patients.

Cytotoxic T lymphocyte-associated antigen-4 (CTLA-4) is a major immune checkpoint that negatively regulates T-cell activation by suppressing T-cell signaling through engagement by B7 (CD80/CD86) ligands (5). As such, CTLA-4 inhibition leads to increased T-cell activation and a reduction in mouse tumor size (6). In contrast to activated T cells expressing CTLA-4 after activation, regulatory T (Treg) cells constitutively express CTLA-4 and play a significant role in tumor tolerance through surface/intracellular CTLA-4 and its soluble counterpart (7). Therefore, targeting CTLA-4 affects both activated T and Treg cells. While anti-CTLA-4 therapy with the monoclonal antibody ipilimumab has improved the overall survival of metastatic melanoma patients (8) compared to anti-PD-1 therapy, a smaller percentage of patients benefited from anti-CTLA-4 treatment, and more immunotherapy-related adverse events were reported (9–11). To better understand the challenges of targeting CTLA-4 and close the fundamental knowledge gap, researchers have attempted to modify anti-CTLA-4 therapy and investigate TME (12).

Immune-related factors, including tumor-infiltrating lymphocytes and immune-related gene signatures, have been utilized to help determine prognosis and response to immunotherapy in melanoma patients (13). However, unlike PD-1 and PD-L1 expression, the expression of CTLA-4 in the TME has rarely been studied. Recent studies have shown that pre-treatment levels of *CTLA4* expression in tumor samples are associated with clinical benefits from anti-CTLA-4 immunotherapy in metastatic melanoma patients (14). Another study found that pre-treatment levels of *CTLA4* promoter methylation (*mCTLA4*) in the tumors inversely correlate with *CTLA4* mRNA expression and that low *mCTLA4* levels are associated with response to anti-PD-1/CTLA-4 therapy (15, 16), suggesting that tumors with higher *CTLA4* expression are associated with clinical benefits, while tumors with lower *CTLA4* expression are not. Interestingly, the study also found that *CTLA4* mRNA expression correlates with patient prognosis even without immunotherapy. These findings suggest that tumors with low *CTLA4* mRNA expression have a poor prognosis and resistance to immunotherapy. Since CTLA-4 expression is dynamically regulated in the TME, these results suggest that tumors with low *CTLA4* mRNA expression may have fewer CTLA-4-positive cells (activated T, Treg, and tumor cells) or a lower amount of *CTLA4* expression per cell. As CTLA-4 is an immune checkpoint that negatively regulates T-cell activation, we aim to investigate the significance of lower CTLA-4 expression in melanoma patients.

The T-cell-intrinsic function of CTLA-4 upregulation has been extensively investigated. On the other hand, Treg cells constitutively express CTLA-4 in physiological conditions; thus, its contribution to Treg cell function has been less studied, particularly in the presence of tumors. Germline depletion of *Ctla4* (17, 18) and Treg-specific deletion of *Ctla4* (19) resulted in severe autoimmunity with lethality, demonstrating the critical role of CTLA-4 in Treg cell function. Based on these findings, the tumors with downregulated CTLA-4 may have enhanced anti-tumor immunity, better prognosis, and greater response to immunotherapy. However, conditional ablation of *Ctla4* in adult mice was reported to confer protection from autoimmune and anti-tumor responses (20), suggesting the Treg-cell-intrinsic function of CTLA-4 to limit their activation and expansion. Therefore, it is also possible that the tumors with downregulated CTLA-4 have reduced anti-tumor immunity, poor prognosis, and resistance to immunotherapy.

In this study, we analyzed the expression of *CTLA4* mRNA in tumor and blood samples from melanoma patients and found a correlation between decreased tumor and blood *CTLA4* and a poorer prognosis in patients with metastatic melanoma. *CTLA4* was downregulated by approximately 70% in Treg cells, resulting in levels similar to those in non-Treg T cells. Mechanistically, we provide evidence that the metastatic melanoma secretome induces post-transcriptional *CTLA4* mRNA instability through the induction of miR-155. Consistent with this, we observed upregulation of miR-155 in Treg cells of metastatic melanoma patients. Functionally, we demonstrated that *CTLA4* expression inhibits the proliferation and suppressive function of human Treg cells. Our findings shed light on the mechanisms underlying the downregulation of *CTLA4* in Treg cells in patients with metastatic melanoma.

## 2 Materials and methods

### 2.1 TCGA and Swedish melanoma tumor datasets

Clinical information on 329 TCGA (TCGA-SKCM cohort) melanoma tumors was obtained from the cBioPortal website (21). Among them, 286 patients had complete pathological information, including 42 cases of primary melanoma and 244 cases of metastatic melanoma. Normalized RNA-seq gene expression profiles (Level 3, RSEM value) of these patients were downloaded from the cBioPortal website (22).

Swedish dataset containing 210 metastatic melanoma tumors was downloaded from the Gene Expression Omnibus (GEO) database with GSE65904, incorporating patient outcomes and relapse-free survival time. A natural log transformation was applied to the processed gene expression data before survival analysis (23).

### 2.2 Characteristics of Australian and US melanoma cohorts for blood analyses

For the AUS cohort, approval for the blood sample study was obtained from the Human Research Ethics Committee of Edith Cowan University (No. 2932) and Sir Charles Gardner Hospital (No. 2007-123). Eligible subjects included 103 healthy donors and 170 melanoma patients recruited in Australia between 2008-2011. All patients were from melanoma clinics in Perth, Western Australia (Medical Oncology Department of Sir Charles Gairdner Hospital and Perth Melanoma Clinic at Hollywood Hospital). Patients comprised 66 women and 104 men, IQR 57-78 years (median age of 66 years), while the aged-matched, healthy cohort from the general population comprised 63 women and 40 men, IQR 32-61.5 years (median age of 45 years). AJCC clinical staging of melanoma patients categorized 71.8% as stages 0-II and 28.2% as stages III-IV.

The US cohort was approved by the Institutional Review Boards of the University of Colorado (COMIRB#05-0309). A total of 263 eligible melanoma patients were recruited from the Cutaneous Oncology Department at the University of Colorado Cancer Center, Aurora, CO. This cohort consisted of 121 women and 142 men, IQR 41-63 years (median age of 53 years). AJCC clinical staging categorized 62.7% as stages 0-II and 37.3% as stages II-IV.

The demographic information of these two cohorts is summarized in **Table 1**. The medical records for each patient were reviewed retrospectively for pertinent past medical history and significant events such as disease progression and death. Local death records were reviewed if survival/mortality information was not determined by chart review. If no proof of death was obtained, the patient was presumed alive. The end of follow-up for AUS and US cohorts was April 14, 2020 and June 19, 2020, respectively. Subjects with non-melanoma-related death, lack of death information, and/or non-cutaneous primary melanoma were excluded from data analysis. Patients with metastatic melanoma of unknown primary origin were included.

**Table 1:** Overview of patient demographic information of AUS and US melanoma patient cohorts.

		AUS melanoma	US Melanoma
Number of Patients		N = 170	N = 263
Sample taken post non-surgical treatment (%)	No	147 (86.5)	217 (82.5)
	Yes	23 (13.5)	46 (17.5)
Sex (%)	Female	66 (38.8)	121 (46.0)
	Male	104 (61.2)	142 (54.0)
Ulceration (%)	No	125 (84.5)	105 (76.1)
	Yes	23 (15.5)	33 (23.9)
Lymph Nodes (%)	No	124 (72.9)	190 (72.2)
	Yes	46 (27.1)	73 (27.8)
Locoregional recurrence (%)	No	157 (92.4)	247 (93.9)
	Yes	13 (7.6)	16 (6.1)
Progression prior to blood draw (%)	No	133 (81.1)	222 (84.4)
	Yes	31 (18.9)	41 (15.6)
Stage at blood draw (%)	0-II	122 (71.8)	165 (62.7)
	III+	48 (28.2)	98 (37.3)
Death (%)	No	136 (80.0)	198 (75.3)
	Yes	34 (20.0)	65 (24.7)
*CTLA4* (median [IQR])		0.00 [0.00, 0.01]	0.01 [0.01, 0.02]
Log *CTLA4* (median [IQR])		-5.63 [-6.00, -5.29]	-4.64 [-5.01, -4.20]
Breslow Thickness (median [IQR])		0.75 [0.29, 2.28]	1.00 [0.52, 2.22]
Years Between Dx and Blood Draw (median [IQR])		1.61 [0.24, 4.41]	0.13 [0.04, 2.19]
Age at Blood Draw (median [IQR])		66.00 [57.00, 78.00]	53.00 [41.00, 63.00]
Years Since Dx (median [IQR])		8.5 [6.38, 11.47]	10.94 [9.2, 11.52]
Years Since Blood Draw (median [IQR])		6.98 [5.46, 7.55]	10.60 [7.68, 11.15]

### 2.3 Blood collection and RNA extraction

PAXgene RNA stabilization tubes (2.5 ml; PreAnalytiX, Hombrechtikon, CH) were used to collect, stabilize, and transport whole blood specimens in a closed evacuated system (24). Following the manufacturer’s protocol, RNA was extracted from the samples using a PAXgene Blood RNA Kit (PreAnalytiX). The quality of RNA was verified on an Agilent 2100 Bioanalyzer (Agilent Technologies, Santa Clara, CA, USA), and the quantity of RNA was determined by a NanoDrop ND-1000 spectrophotometer (Thermo Scientific, Wilmington, DE, USA) before being used in reverse transcription reactions with MMLV reverse transcriptase (Promega, Madison, WI, USA).

### 2.4 Fractionation of blood cell subtypes and RNA extraction

Blood samples to be fractionated were collected from metastatic melanoma patients at the Cutaneous Oncology Department at the University of Colorado Cancer Center, as mentioned previously (24, 25). The desired human immune cells (CD3^+^, CD8^+^, CD14^+^, CD15^+^, CD19^+^, CD45^+^, and CD56^+^) were fractionated from collected blood samples using autoMACS™ separator (Miltenyi Biotec, Auburn, CA, USA). Further isolation of T cell subsets was done using flow cytometric sorting of CD4^+^CD25^−^CD127^hi^ (conventional T cells), CD4^+^CD25^+^CD127^dim^ (Treg cells) and CD8^+^ T cells from blood samples. RNA was immediately extracted from fractionated cells using a Qiagen RNeasy mini kit (Qiagen, Valencia, CA, USA).

### 2.5 Quantitative reverse transcription -PCR

qRT-PCR was performed using Power SYBR Green PCR Master Mix (Applied Biosystems, Foster City, CA) on the MX3000P PCR system (Applied Biosystems). The gene expression was normalized relative to the housekeeping gene *GAPDH*. Primer sequences are listed in **Supplementary Table S1**.

### 2.6 Cell culture of melanoma cell lines

Human primary melanoma cell lines (WM35, WM115, and WM793) and metastatic melanoma cell lines (A375, 1205Lu, and HS294T) were obtained from the American Type Culture Collection (Manassas, VA) and cultured in RPMI 1640 (Thermo Scientific, Rockford, IL, USA) supplemented with 10% fetal bovine serum (Gemini Bioproducts, West Sacramento, CA, USA), 100 IU/ml penicillin-100 μg/ml streptomycin (Mediatech, Manassas, VA, USA) at 37°C and 5% CO_2_ in the incubator. Melanoma-conditioned media (MCM) was obtained from culture supernatants of human melanoma cells after 24 h of cultivation in OptiMEM (Life Technologies, Grand Island, NY, USA) and centrifuged at 210 × g for 5 min (26). These cell lines have been authenticated using the short tandem repeat (STR) fingerprinting by the Barbara Davis Center Bioresource Core at the University of Colorado Anschutz Medical Campus. Cells were regularly monitored for mycoplasma contamination using PCR.

### 2.7 Isolation of human PBMCs for cell culture and *CTLA4* quantification

Blood from healthy donors was collected at the Children’s Hospital Blood Donor Centre in Aurora, CO, USA, approved under COMIRB#17–0110. We isolated human peripheral blood mononuclear cells (PBMCs) using the density gradient separation media-Histopaque 1077 (Sigma-Aldrich, St. Louis, MO, USA) and evaluated the effect of melanoma secretome on *CTLA4* expression. The isolated PBMCs were cultured with 50% culture media (RPMI 1640 + 10% fetal bovine serum) and 50% MCM from human primary melanoma cell lines (WM115, WM35, and WM793) and metastatic melanoma cell lines (1205Lu, A375, and HS294T) for 24 h. *CTLA4* expression in control media- and MCM-treated PBMCs was quantified by qRT-PCR as mentioned above. Primer sequences are listed in **Supplementary Table S1**.

### 2.8 Flow cytometry analysis

Treg cells (CD4^+^CD25^+^CD127^dim^) were isolated from human healthy donor PBMCs using a CD4^+^CD25^+^CD127^dim^ Treg cell Isolation kit (Miltenyi Biotech, San Diego, CA, USA) according to the manufacturer’s instructions. Isolated human Treg cells were cultured in 50% lymphocyte cell culture media (RPMI 1640 supplemented with 10% fetal bovine serum, 0.1% β-mercaptoethanol (#21985023; Thermo Scientific, Rockford, IL, USA), 1% non-essential amino acids (#25025; Mediatech, Manassas, VA, USA), and 2 mM glutamine (#35050061; Thermo Scientific, Rockford, IL, USA)) with 50% MCM from 1205Lu cells. *CTLA4* and *FOXP3* expression in control media- and MCM-treated Treg cells were quantified by qRT-PCR. Primer sequences are listed in **Supplementary Table S1**.

Similarly, to evaluate the effect of melanoma TME on CTLA-4 and FOXP3 expression in Treg cells at the protein level, isolated Treg cells were cultured with and without 50% MCM for 48 h. Cultured Treg cells were stained for cell surface CTLA-4 and intracellular FOXP3 using the respective flow antibodies (BioLegend, San Diego, CA, USA) and FOXP3 Cytoperm/Cytofix staining kit (BD Pharmingen, San Diego, CA, USA) and analyzed using flowcytometry-Gallios 561 (Beckman Coulter, Indianapolis, IN, USA).

Next, to evaluate the effect of MCM or CTLA-4 downregulation in Treg cells on their proliferation and function, we assessed the proliferation of isolated Treg cells treated with 50% MCM or siRNA transfected or control Treg cells using an XTT assay. Briefly, Treg cells transfected with *CTLA4*-siRNA or control-siRNA were seeded into 96-well plates and cultured for 72 h. In another setting, Treg cells were seeded into 96-well plates and treated with or without 50% MCM in lymphocyte cell culture media for 72 h. Cell proliferation was assayed daily by adding XTT compound (2, 3-bis (2-methoxy-4-nitro-5-sulphophenyl)-2H-tetrazolium-5-carboxanilide) (#X6493; Thermo Scientific, Rockford, IL, USA) followed by measuring absorbance at 450 nm in a microplate reader (Bio Tek, Winooski, VT, USA).

Likewise, T cell suppression assay was conducted using the CFSE- (#C34554; Thermo Scientific, Rockford, IL, USA) labeled T conventional (Tconv) cells (CD4^+^CD25^-^CD127^hi^) in the presence of Treg cells subjected to various treatments. Isolated Treg cells were cultured with 50% MCM + 50% lymphocyte culture media (MCM-treated Treg cells) or 100% lymphocyte culture media (control Treg cells) for 48 h. CFSE-labeled Tconv cells were co-cultured with MCM-treated Treg cells or control Treg cells at ratios (1:1, 1:5, and 1:10; Treg: Tconv) in lymphocyte culture media with CD3/CD28 beads (1:2 beads: Tconv) (#130-095-345; Miltenyi Biotech, San Diego, CA, USA) and recombinant human IL-2 (100 IU/ml) (#202-IL-010/CF; R&D Systems, Minneapolis, MN, USA) for 72 h. In another setting, CFSE-labeled Tconv cells were co-cultured with siRNA-transfected Treg cells (*CTLA4*-siRNA or control-siRNA) for 72 h, as mentioned above. The dilution of CFSE in CFSE-labeled Tconv cells was analyzed using the flowcytometry-Gallios 561 (Beckman Coulter, Indianapolis, IN, USA).

### 2.9 miRNA bioinformatics tools

Three miRNA prediction bioinformatics tools, TargetScan (27) (https://www.targetscan.org/vert_80/), miRanda (28) (http://www.microrna.org/), and PicTar (29)(http://pictar.bio.nyu.edu/) were used to predict the potential miRNAs targeting the *CTLA4* mRNA. TargetScan was used to obtain the predicted complementary 3’UTR sequence of *CTLA4* to the seed sequence of miR-155 using (27).

### 2.10 miRNA quantification

miRNAs were extracted from the cells using the mirVana miRNA isolation kit (Thermo Fisher Scientific, Waltham, MA, USA) following the manufacturer’s instructions. RT-PCR and qPCR were performed using miRNA-specific TaqMan™ MicroRNA assay kits for miRs-9, -34c, -142, -145, -155, -324, and -449 (Thermo Fisher Scientific, Waltham, MA, USA). U6 snRNA was used as an internal control to normalize miRNA expression (30).

### 2.11 Transfection of siRNAs, miR-155 mimics, and inhibitors

Human Treg cells were isolated from healthy donor PBMCs and transfected with 50 nM siRNA (a mixture of two preselected siRNAs; Horizon, Boulder, CO, USA) targeting argonaute-2 (*AGO2*), *CTLA4*, 50 nM miRNA mimics (Horizon), miRNA inhibitors (Horizon) targeting miR-155, or their corresponding non-target control using the P3 transfection buffer (Lonza, Hayward, CA, USA) with the protocol EO-104 on the Lonza 4D instrument (Lonza, Hayward, CA, USA). Transfected Treg cells were cultured for 24 h in the lymphocyte culture media with or without 50% MCM from 1205Lu cells. Then, RNA and miRNAs were isolated from cultured Treg cells and quantified for the expression of *AGO2*, *CTLA4*, *FOXP3*, miR-155, U6 snRNA, and *GAPDH*, as mentioned (31). The primers are listed in **Supplementary Table S1**.

### 2.12 Analysis of CTLA-4 protein expression in immune cell subtypes

We used the publicly available Cytometry by Time of Flight (CyTOF) data to evaluate the protein expression of CTLA-4 in blood samples of healthy donors (n = 5) and metastatic melanoma patients (n = 10). The clinical information of the cohort (n = 15) (#2 dataset) has been described previously (32). We accessed the normalized CyTOF data from Flow Repository using repository ID: FR-FCM-ZY34 (33) and analyzed CTLA-4 expression in CD45^+^, CD4^+^, CD8^+^, and CD4^+^CD25^+^CD127^-^ immune cell subtypes using the FlowJo software (BD Company, Ashland, OR, USA).

### 2.13 Statistical analysis

Statistical univariable analysis of melanoma-specific survival was determined using R 4.2.0 using Package ‘survival’ version 3.5-0. An optimal *CTLA4* expression level cut-point associated with overall survival (OS) was identified by incrementing over the range of expression values and dichotomizing patients into low and high expressers of natural log transformed *CTLA4* values that were higher or lower than the cut-point, with the cut-point that provided the strongest separation in OS was selected based on the log-rank test. Cut-points were assessed in increments of 0.01, and any that resulted in a large group imbalance (< 25% of patients in one group) were not evaluated.

The overall survival of melanoma patients with low and high expressers of *CTLA4* was calculated using the log-rank test and Kaplan-Meier (K-M) plot in the total, primary (stages 0 - II), and malignant (stages III – IV+) population of TCGA, Swedish AUS, and USA melanoma cohorts. For survival analyses, death was considered the endpoint, and overall survival was defined as the interval from primary diagnosis to death.

Since sex and age are known to dictate melanoma patient prognosis (34), Cox proportional hazards (Cox PH) regression was used for multivariable analysis. Cox PH analyses were performed with dichotomized (based on optimal cut-point) patient population and continuous *mRNA* expression data (natural log-transformed) after adjusting for age and sex, and disease stage. Two-sided P values < 0.05 were considered statistically significant, and we made no adjustments for multiple comparisons.

Other experimental results are derived from at least 3 independent experiments. The numerical data are expressed as mean ± SEM. Prism (version 7.0d) software (GraphPad Software, Inc, La Jolla, CA, USA) calculated the differences between the groups by one-way ANOVA with Bonferroni’s or Dunnett’s post-tests. A value *p* < 0.05 was considered significant.

## 3 Results

### 3.1 Correlation between lower tumor *CTLA4* mRNA levels and worse prognosis in metastatic melanoma patients across two cohorts

**Figure 1:**
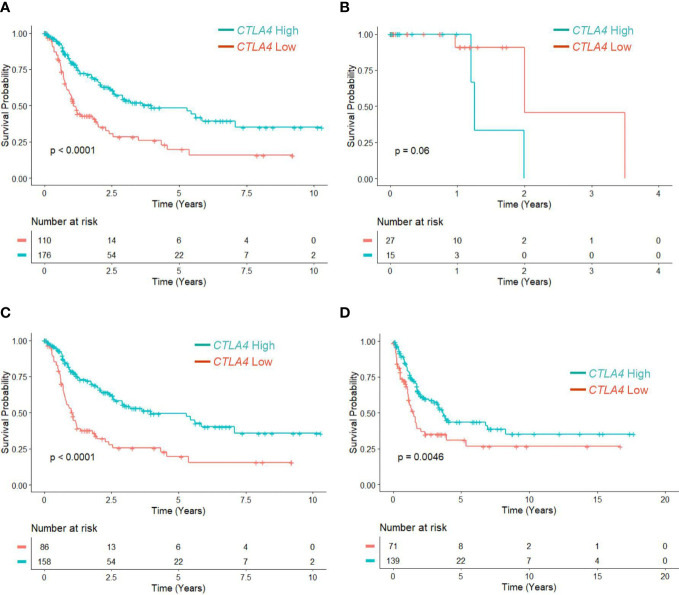
The prognostic value of *CTLA4* expression in tumor samples of melanoma patients from TCGA and Swedish cohorts. **(A-C)** Kaplan-Meier survival analyses of TCGA melanoma patients stratified as low and high based on the optimal cut-point of tumor *CTLA4* expression levels in whole melanoma patient population (n = 286) **(A)**, primary melanoma population (n = 42) **(B)**, and metastatic melanoma population (n = 244) **(C)**. **(D)** Kaplan-Meier survival analyses of the Swedish cohort of metastatic melanoma (n = 210). The significance of overall survival between low and high *CTLA4*-expressing patients was calculated by log-rank test.

Since the association of tumor *CTLA4* expression with OS in melanoma patients has not been well characterized, we first analyzed this relationship in TCGA melanoma patients. Patients were divided into low (n = 110) and high (n = 176) *CTLA4* expressers based on the optimal cut-point (log –3.7) of *CTLA4* mRNA expression. The K-M survival curve analysis showed that patients with low *CTLA4* expression were associated with worse OS than patients with high *CTLA4* expression (*p* = 0.0001) (**Figure 1A**), similar to the findings from Goltz et al. (15). Further analysis showed that among these patients, not those with primary melanoma (n = 42) (**Figure 1B**) but those with metastatic melanoma (n = 244) had a significant association between low *CTLA4* expression and worse OS (*p* = 0.0001) (**Figure 1C**).

To verify this new finding, we performed a survival analysis with the log-rank test on another publicly available dataset of metastatic melanoma tumors from the Swedish cohort (23). Patients were segregated as low (n = 71) and high (n = 139) *CTLA4* expressers using the optimized cut-point (log –5.17). The results showed that downregulated *CTLA4* expression was associated with worse OS in the Swedish dataset of metastatic melanoma patients (*p* = 0.0046) (**Figure 1D**), consistent with the observations in the TCGA dataset. These findings collectively indicate that different from previously reported data, *CTLA4* is not upregulated in metastatic melanoma, and downregulated *CLTA4* correlates with poor prognosis in those metastatic melanoma patients.

### 3.2 Correlation between lower blood *CTLA4* mRNA levels and worse prognosis in metastatic melanoma patients across two cohorts

**Figure 2:**
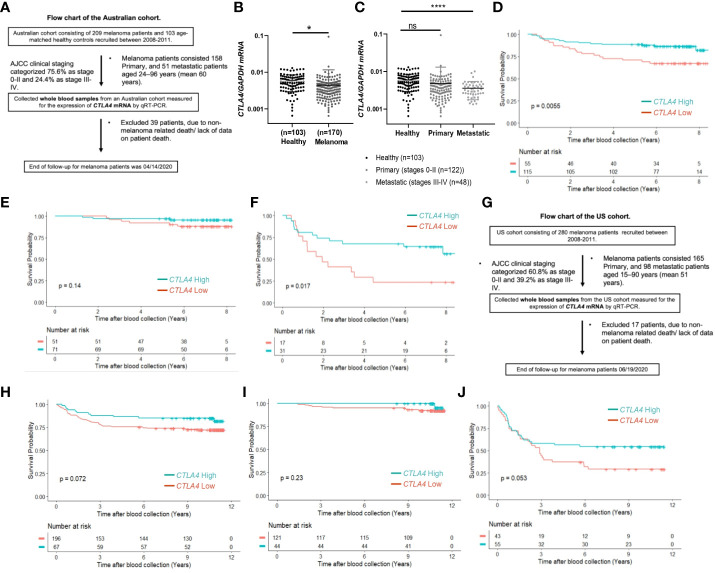
The prognostic value of *CTLA4* expression in blood samples of melanoma patients from AUS and US cohorts. **(A-F)** AUS cohort data. **(A)** The flow chart of the AUS cohort. **(B, C)** qRT-PCR analysis of blood *CTLA4* mRNA expression in healthy donors (n = 103) and melanoma patients (n = 170) (**B**), and healthy donors (n = 103), primary melanoma (n = 122), and metastatic melanoma (n = 48) **(C)**. *CTLA4* mRNA expression levels were normalized to *GAPDH* expression. Data are expressed as the mean ± SEM, ns: not significant, *p < 0.05, and ****p < 0.0001. **(D–F)** Kaplan-Meier survival analyses of AUS melanoma patients stratified according to the optimal cut-point of *CTLA4* mRNA expression in blood samples of all melanoma patients (n = 210) **(D)**, primary (stages 0-II) melanoma patients (n = 122) **(E)**, and metastatic (stages III-IV+) melanoma patient (n = 48) **(F)**. **(G-J)** US cohort data. **(G)** The flow chart of the US cohort. **(H-J)** Kaplan-Meier survival analyses of the US cohort with all melanoma patients (n = 263) **(H)**, primary (stages 0-II) melanoma patients (n = 165) **(I)**, and metastatic (stages III-IV+) melanoma patients (n = 98) **(J)**. The significance of overall survival between low and high *CTLA4*-expressing patient groups was calculated by log-rank test.

Although tumor *CTLA4* levels are associated with prognosis in patients with metastatic melanoma, tumor *CTLA4* expression levels are dynamically regulated by various factors and are technically challenging to test. Because CTLA-4 is expressed in immune cells such as Treg cells and activated T cells, we wondered whether we could observe similar or different trends in blood samples of melanoma patients. We collected blood samples from the AUS and US cohorts and analyzed them for *CTLA4* expression. **Figure 2A** explains the flow chart of blood sample collection from the AUS cohort. An overview of *CTLA4* expression data in the AUS cohort is shown in **Supplementary Table S2**. The AUS cohort comprised healthy donors (n = 103) and melanoma patients (n = 209, including 158 primary and 51 metastatic cases). Among the 209 patients, 36 primary and 3 metastatic patients were excluded due to non-melanoma-related death or lack of data on patient death, and the remaining 170 patients and 103 healthy donors were included for data analysis. The results showed that the expression levels of blood *CTLA4* in melanoma patients were significantly lower than those in healthy donors (*p* = 0.037) (**Figure 2B**). This significant difference was due to the significant downregulation of *CTLA4* in metastatic melanoma patients (stages III-IV, n = 48) (*p* = 0.0001) but not in primary melanoma patients (stages 0-II, n = 122) (**Figure 2C**). As expected, K-M plot analysis showed that OS worsened in melanoma patients as the disease progressed (**Supplementary Figure 1**), suggesting that blood *CTLA4* levels decline as melanoma progresses and the declined *CTLA4* could correlate with poor prognosis of patients.

Therefore, we categorized 170 melanoma patients as high and low expressers of *CTLA4* based on the optimized cut-point for survival analysis. As shown in **Figure 2D**, survival curve analysis with an optimal cut point (log –5.91) showed that patients with low blood *CTLA4* expression levels (n = 55) were significantly associated with worse OS compared to those patients with high blood *CTLA4* expression levels (n = 115) (*p* = 0.0055). Further analysis showed that among these melanoma patients, not those with primary melanoma (stages 0-II, n = 122, *p* = 0.14) (**Figure 2E**), but those with metastatic melanoma (stages III-IV, n = 48) had a significant association between low blood *CTLA4* expression and worse OS based on the optimal cut point (log –5.93) (*p* = 0.017) (**Figure 2F**). These data indicate that low tumor and low blood *CTLA4* expression levels are associated with worse OS in metastatic melanoma patients.

As sex and age are well-known determinants of melanoma patient prognosis (34), a multivariable Cox PH analysis was applied, including the confounding effects of sex, age, and stage to compare the cumulative mortality in two log-*CTLA4* level subgroups based on the cut-point in the respective AUS cohorts (**Table 2**). AUS patients with high (≥–5.91) *CTLA4* levels experienced a 47% lower risk of death (HR = 0.53 [95% CI: 0.27 – 1.06], *p* = 0.074) than patients with lower (<–5.91) *CTLA4*. Specifically, in AUS metastatic melanoma cohort, patients with high (≥–5.93) *CTLA4* experienced a better prognosis and a 55% lower risk of death (HR = 0.45 [95% CI: 0.2 – 1.02], *p* = 0.057) than those with lower (<–5.93) *CTLA4*. In contrast, in the primary melanoma cohort, no assessment was made as the hazard ratio (HR) was not supported by broader CI. These statistical analyses demonstrate that low blood *CTLA4* levels are associated with worse melanoma patient prognosis irrespective of age and sex in this cohort.

**Table 2:** Association between survival, log *CTLA4* cut-point, melanoma stage, age, and sex by multivariable Cox PH model for AUS patients (n = 170, 34 events), primary melanoma (n = 122, 9 events), and metastatic melanoma (n = 48, 25 events).

Log(*CTLA4*)	AUS melanoma population			AUS primary melanoma			AUS metastatic melanoma		
	<-5.91 vs ≥-5.91			<-5.76 vs ≥-5.76			<-5.93 vs ≥-5.93		
	HR	95% CI	*p*	HR	95% CI	*p*	HR	95% CI	*p*
Stage (III+ vs. 0-II)	7.94	3.54 - 17.81	<0.001	–	–	–	–	–	–
Log(*CTLA4*)	0.53	0.27 - 1.06	0.074	0.29	0.07 - 1.17	0.082	0.45	0.2 - 1.02	0.057
Sex (males vs. females)	2.44	0.99 - 6.05	0.053	5.32	0.98 - 28.88	0.053	2.12	0.71 - 6.28	0.177
Age (years)	0.99	0.97 - 1.02	0.566	0.92	0.87 - 0.98	0.012	1.00	0.98 - 1.03	0.867

To verify our new finding in blood *CTLA4* levels and their prognostic values, we performed another study using the US melanoma cohort, shown in the flow chart (**Figure 2G**). Similar to the AUS cohort survival analysis, US cohort melanoma patients (n = 263) were segregated as *CTLA4* high and low expressers based on the optimized cut point (log –4.21) for survival analysis. Similar to the AUS cohort, univariable survival curve analysis showed that patients with low blood *CTLA4* expression (n = 196) had a worse prognosis compared to those with high blood *CTLA4* levels (n = 67) (*p* = 0.072) (**Figure 2H**). Similarly, further analysis showed that not those with primary melanoma (stages 0-II, n = 165, *p* = 0.23) (**Figure 2I**) but those with metastatic melanoma (stages III-IV, n = 98) had an association between low blood *CTLA4* expression and worse OS based on the optimal cut point (log –4.73); however, it was not statistically significant (*p* = 0.053) (**Figure 2J**).

Cox PH analysis was also applied to compare the cumulative death rates in two log-*CTLA4* level subgroups based on the cut-point in the respective US melanoma cohort (**Table 3**). US melanoma patients with higher (≥–4.21) *CTLA4* experienced a 35% lower risk of death (HR = 0.65 [95% CI: 0.34 - 1.26], *p* = 0.202) than those with lower (<–4.21) *CTLA4*. Likewise, in the US metastatic melanoma cohort, patients with higher (≥–4.73) *CTLA4* experienced better prognosis and 24% lower risk of death (HR = 0.76 [95% CI: 0.43 - 1.35], p = 0.353) than those with lower (<–4.73) *CTLA4*. However, in the primary melanoma cohort, no assessment was made as broader CI did not support HR.

**Table 3:** Association between survival, log CTLA4 cut-point, melanoma stage, age, and sex by multivariable Cox PH model for US patients (n = 263, 65 events), primary melanoma (n = 165, 10 events), and metastatic melanoma (n = 98, 55 events).

Log(*CTLA4*)	US melanoma population			US primary melanoma			US metastatic melanoma		
	<-4.21 vs ≥-4.21			<-4.24 vs ≥-4.24			<-4.73 vs ≥-4.73		
	HR	95% CI	*p*	HR	95% CI	*p*	HR	95% CI	*p*
Stage (III+ vs. 0-II)	12.8	6.48 - 25.29	<0.001	–	–	–	–	–	–
Log(*CTLA4*)	0.65	0.34 - 1.26	0.202	0.37	0.05 - 2.98	0.353	0.76	0.43 - 1.35	0.353
Sex (males vs. females)	1.45	0.83 - 2.53	0.197	2.16	0.42 - 11.01	0.354	1.29	0.71 - 2.35	0.401
Age (years)	1.03	1.01 - 1.05	0.001	1.07	1.02 - 1.13	0.010	1.02	1 - 1.05	0.040

The results from the AUS cohort confirm that lower blood *CTLA4* levels are associated with worse prognosis in metastatic melanoma patients. Although the results from the US cohort did not reach statistical significance, similar trends were observed in patients. Furthermore, the data from the AUS cohort indicate that blood *CTLA4* levels determine the melanoma patient prognosis independent of age and sex.

### 3.3 Treg cells contribute to reduced blood *CTLA4* mRNA levels in metastatic melanoma patients

**Figure 3:**
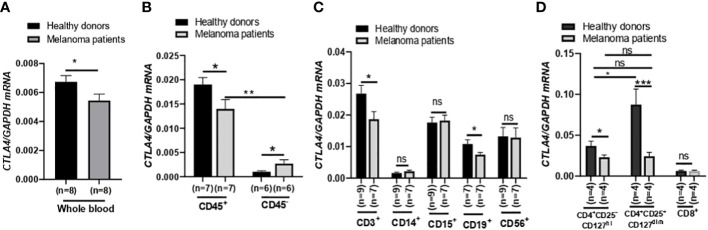
Expression of *CTLA4* mRNA and CTLA-4 protein in blood samples of healthy donors and metastatic melanoma patients. **(A)** qRT-PCR analysis of *CTLA4* expression in whole blood of healthy donors and metastatic melanoma patients (n = 8 each). **(B–D)** qRT-PCR analysis of *CTLA4* expression in fractionated CD45^+^ and CD45^-^ cells from healthy donors and metastatic melanoma patients (n = 8 each) **(B)**, T cells (CD3^+^), monocytes/macrophages (CD14^+^), granulocytes (CD15^+^), B cells (CD19^+^), and natural killer cells (CD56^+^) from healthy donors (n = 9) and metastatic melanoma patients (n = 7) **(C)** and CD4^+^CD25^−^CD127^hi^ (conventional T cells: Tconv cells), CD4^+^CD25^+^CD127^dim^ (Treg cells), and CD8^+^ T cells from healthy donors and metastatic melanoma patients (n = 4 each) **(D)**. *CTLA4* expression was normalized using *GAPDH* as an internal control. Representative data are shown and expressed as the mean ± SEM, ns, not significant, *p < 0.05, ** *p* < 0.01, and *** *p* < 0.001.

After finding that blood *CTLA4* expression was associated with melanoma patients’ OS, we aimed to investigate which specific blood cell subtypes were responsible for the downregulation of *CTLA4* expression. To test this, we analyzed blood samples from a different cohort of healthy donors and metastatic melanoma patients in the US (**Figure 3**). We first confirmed that levels of *CTLA4* were downregulated in whole blood samples from melanoma patients compared to healthy donors (**Figure 3A**). Along with immune cells, tumor cells also express *CTLA4*, induced by tumor cell-intrinsic b-catenin signaling (35). Because whole blood samples contain circulating tumor cells, we first fractionated the samples into CD45^+^ (leukocytes) and CD45^-^ (erythrocytes, platelets, and non-immune cells) fractions (**Figure 3B**). *CTLA4* was almost exclusively expressed in CD45^+^ cells but rarely detected in CD45^-^ cells in healthy donors. This trend was similar in metastatic melanoma patients, while *CTLA4* levels in CD45^-^ cells were almost doubled compared to healthy donors. Similar to whole blood samples, *CTLA4* levels were downregulated in CD45^+^ cells from melanoma patients compared to healthy donors.

We then compared *CTLA4* expression in various circulating immune cell fractions, including CD3^+^ (T cells), CD14^+^ (monocytes), CD15^+^ (granulocytes), CD19^+^ (B cells), and CD56^+^ (neutrophils), from the two groups. While *CTLA4* expression was not significantly different in CD14^+^, CD15^+^, and CD56^+^ cells between the two groups (**Figure 3B**), the levels were significantly downregulated by approximately 30% in both CD3^+^ and CD19^+^ cells from metastatic melanoma patients compared to healthy donors (**Figure 3C**).

Since *CTLA4* expression levels were much higher in CD3^+^ cells than in CD19^+^ cells, we further fractionated CD3^+^ cells and analyzed *CTLA4* expression in Tconv cells (CD4^+^CD25^−^CD127^hi^), Treg cells (CD4^+^ CD25^+^ CD127^dim^), and CD8^+^ T cell subsets (**Figure 3D**). As expected, *CTLA4* was highly expressed in the Treg cell subset but barely expressed in the CD8+ T cell subset in healthy donors. However, the expected *CTLA4* upregulation was not observed in the Treg cell subset of metastatic melanoma patients. In fact, *CTLA4* expression was downregulated by approximately 70% in Treg cell fractions of metastatic melanoma patients’ blood compared to healthy donors. The *CTLA4* expression levels in the Treg cell subset of metastatic melanoma patients were similar to those in the Tconv cell subset of metastatic melanoma patients and were even lower than those in the Tconv cells of healthy donors. These results demonstrate that *CTLA4* expression levels in Treg cells from metastatic melanoma patients are severely downregulated or almost abolished, suggesting an active transcriptional or post-transcriptional inhibition of *CTLA4* mRNA induction in human Treg cells.

### 3.4 Human metastatic melanoma secretome upregulates *FOXP3* but downregulates *CTLA4* in human treg cells and enhances their proliferation and suppressive function

**Figure 4:**
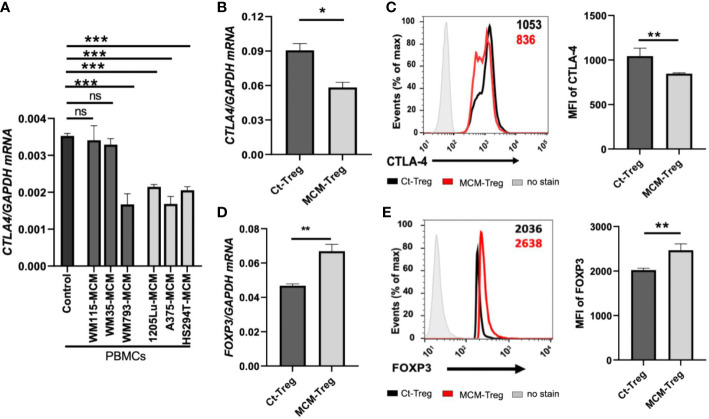
The effects of MCM on CTLA-4 and FOXP3 expression in PBMCs and human Treg cells. **(A)** qRT-PCR analysis of *CTLA4* in PBMCs cultured for 24 h in control media or 50% control media + 50% MCM from human primary melanoma cell lines (WM35, WM115, and WM793) or metastatic melanoma cell lines (A375, 1205Lu, and HS294T). **(B)** The qRT-PCR quantification of *CTLA4* mRNA expression in human Treg cells cultured in control media (Ct-Treg) or 50% control media + 50% 1205Lu-MCM (MCM-Treg) for 24 h. The expression of *CTLA4* was normalized using *GAPDH* as an internal control. **(C)** The flow cytometry analysis of CTLA-4 surface protein in Treg cells cultured for 48 h. Treg cells not stained with the antibodies are used as a control (“No stain”). Representative histogram with mean fluorescent intensity (MFI) in black (Ct-Treg) and red (MCM-Treg) (left panel) and the MFI analysis (right panel). **(D)** The qRT-PCR quantification of *FOXP3* mRNA expression in Ct-Treg and MCM-Treg cultured for 24 h. **(E)** The flow cytometry analysis of FOXP3 protein expression in Treg cells cultured for 48 h. Representative histogram with MFI in black (Ct-Treg) and red (MCM-Treg) (left panel) and the MFI analysis (right panel). Representative data are shown and expressed as the mean ± SEM (n = 3). ns: not significant, *p < 0.05, ** *p* < 0.01, and *** *p* < 0.001.

We aimed to understand the mechanisms underlying the downregulation of *CTLA4* expression in human Treg cells. As T-cell function is influenced by receptor signaling through T-cell receptors and cytokines, we hypothesized that the tumor cell secretome downregulates *CTLA4* expression in human Treg cells. To test this hypothesis, we analyzed the effects of human melanoma cell secretomes on *CTLA4* expression in human PBMCs by culturing them with MCM obtained from various human melanoma cell lines. qRT-PCR analysis showed that the expression of *CTLA4* was significantly downregulated in PBMCs treated with MCM from metastatic melanoma cell lines (1205Lu, A375, and HS294T) (**Figure 4A**). In contrast, MCM from 2 out of three primary melanoma cell lines tested (WM115 and WM35) did not inhibit the expression of *CTLA4* in PBMCs. These data suggest that tumor cell secretome from metastatic melanoma cells mediates the downregulation of *CTLA4* in immune cells.

FOXP3 is a known transcriptional activator of *CTLA4* expression in Treg cells (19). Therefore, we tested whether melanoma cell secretome-mediated *CTLA4* downregulation is regulated at the transcriptional level through FOXP3 in Treg cells. Isolated human Treg cells were cultured with or without 50% 1205Lu-MCM for 24 h. qRT-PCR and flow cytometry analyses confirmed that CTLA-4 expression was downregulated at mRNA (**Figure 4B**) and protein levels (**Figure 4C**). However, the mRNA and protein expression of FOXP3 was significantly upregulated in human Treg cells treated with MCM for 24 or 48 h (**Figures 4D, E**, respectively), suggesting that downregulation of *CTLA4* mRNA in Treg cells occurs at the post-transcriptional level. The time-course experiments showed that the exposure to MCM led to *CTLA4* downregulation in Treg cells by 12 h, and this effect persisted at least 94 h (**Supplementary Figure 2**).

**Figure 5:**
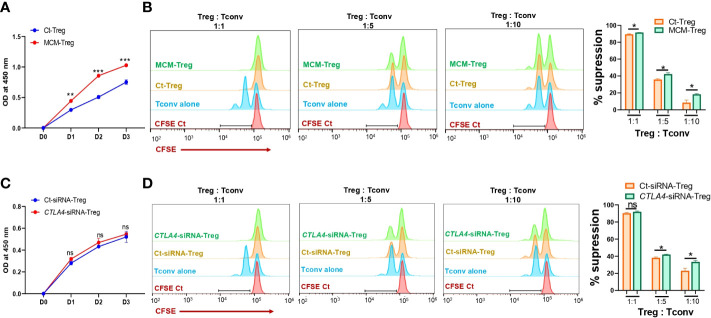
The effects of MCM and CTLA-4 on human Treg cell proliferation and functionality. **(A)** XTT colorimetric assessment of the proliferation of human Treg cells cultured in control media (Ct-Treg, blue) or 50% control media + 50% 1205Lu-MCM (MCM-Treg, red) for 72 h. **(B)** The flow cytometry analysis of CFSE dilution in CFSE-labeled Tconv cells co-cultured with control Tregs cells or MCM-treated Treg cells at different ratios (1:1, 1:5, and 1:10, Treg: Tconv) or CFSE-labelled Tconv cells alone in lymphocyte culture media with CD3/CD28 beads and rhIL-2 for 72 h. The representative histogram in orange (Ct-Treg), green (MCM-Treg), blue (Tconv alone), and red (CFSE-labeled Tconv on Day 0) (left panel) and percent suppression (right panel). **(C)** XTT colorimetric assessment of the proliferation of human Treg cells transfected with control siRNA (blue) or *CTLA4* siRNA (red) for 72 h. **(D)** The flow cytometry analysis of CFSE dilution in CFSE-labeled Tconv cells co-cultured with control-siRNA transfected Tregs cells or *CTLA4*-siRNA transfected Treg cells at different ratios (1:1, 1:5, and 1:10, Treg: Tconv) or CFSE-labeled Tconv cells alone in lymphocyte culture media with CD3/CD28 beads and rhIL-2 for 72 h. The representative histogram in orange (Ct-siRNA-Treg), green (*CTLA4*-siRNA-Treg), blue (Tconv alone), and red (CFSE-labeled Tconv on Day 0) (left panel) and percent suppression (right panel). Data are expressed as the mean ± SEM (n = 3). ns, not significant, *p < 0.05, ** *p* < 0.01, and *** *p* < 0.001.

Since MCM induces downregulation of CTLA-4 and upregulation of FOXP3 in Treg cells, we further assessed the effect of MCM on Treg cell proliferation and suppressive function. Human Treg cells treated with 50% MCM showed enhanced proliferation (**Figure 5A**) and increased suppressive function compared to control Treg cells without MCM treatment (**Figure 5B**; **Supplementary Figure 3A**). These results suggest that MCM augments Treg cell suppressive function by inducing Treg cell proliferation (as shown in **Figure 5A**) or promoting *FOXP3* expression and functionality in Treg cells (as shown in **Figures 4D, E**). As MCM upregulates *FOXP3* while downregulating *CTLA4*, we assessed the effect of downregulated *CTLA4* in Treg cells on their proliferation and suppressive function. We generated *CTLA4*-silenced Treg cells using siRNA (**Supplementary Figure 3**). As shown in **Figure 5C**, *CTLA4* knockdown did not affect Treg cell proliferation. However, Treg cells with *CTLA4* knockdown significantly suppressed CFSE-stained Tconv cell proliferation compared to control Treg cells (**Figure 5D**; **Supplementary Figure 4B**). These data suggest that *CTLA4* plays a role in decreasing Treg cell suppressive function.

### 3.5 *CTLA4* in human treg cells is post-transcriptionally downregulated by miRNAs induced by melanoma secretome

**Figure 6:**
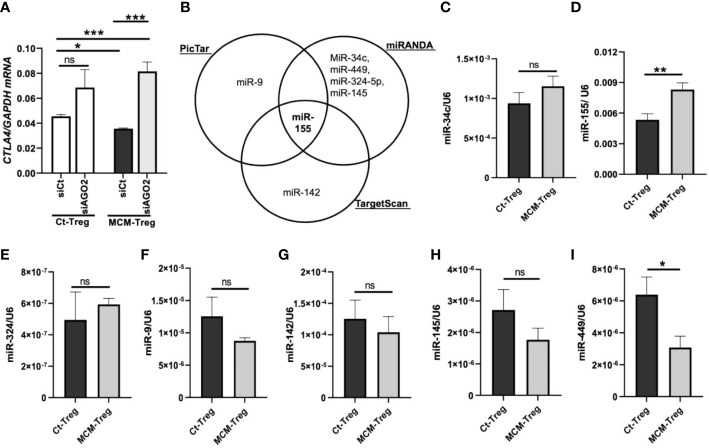
MicroRNAs regulation of *CTLA4* expression in human Treg cells. **(A)** qRT-PCR analysis of *CTLA4* expression in human Treg cells transfected with 50 nM control siRNA (siCt) or AGO2 siRNA (siAGO2) and subsequently cultured for 24 h in control media (Ct-Treg) or 50% control media + 50% 1205Lu-MCM (MCM-Treg). The expression of *CTLA4* was normalized using *GAPDH* as an internal control. **(B)** Venn diagram of predicted miRNAs targeting the 3’UTR of *CTLA4* mRNA from three bioinformatics tools (PicTar, miRanda, and TargetScan). **(C-I)** Quantitative analysis of mature miR-34c **(C)**, miR-155 **(D)**, miR-324 **(E)**, miR-9 **(F)**, miR-142 **(G)**, miR-145 **(H)**, and miR-449 **(I)** expression in Ct-Treg and MCM-Treg cells for 24 h. The expression of miRNAs were normalized using U6 snRNA as an internal control. Representative data are shown and expressed as the mean ± SEM (n = 3). ns, not significant, *p < 0.05, ** *p* < 0.01, and *** *p* < 0.001.

We investigated the mechanisms of *CTLA4* downregulation by melanoma cell secretome in human Treg cells. Given the opposing effects of the secretome on *CTLA4* and *FOXP3* expression, we hypothesized that the secretome-mediated downregulation of *CTLA4* occurs downstream of gene transcription. To test this, we knocked down *AGO2*, a major component of the miRNA-induced silencing complex, and examined its effect on *CTLA4* gene expression. AGO-2 typically binds to the 3′UTR of cytosolic mRNA targets, resulting in mRNA degradation (36). Human Treg cells were transfected with *AGO2* siRNA and cultured in 50% 1205Lu-MCM for 24 h. As shown in **Figure 6A**, silencing *AGO2* increased *CTLA4* expression in Treg cells, and this increase was significant in the presence of MCM, suggesting that RNA interference is likely involved in the instability of *CTLA4* mRNA in secretome-treated Treg cells. Therefore, the *CTLA4* downregulation observed in melanoma cell secretome-treated Treg cells was achieved through miRNA-mediated gene silencing at the post-transcriptional level.

To identify miRNAs targeting the *CTLA4* 3′UTR, we first performed an in silico screening of putative miRNA using three bioinformatics tools (PicTar, TargetScan, and miRanda). We selected several potential miRNAs, including miRs-9, -34c, -449, -324-5p, -145, -142, and -155, based on their binding sites spanning the *CTLA4* 3′UTR (**Figure 6B**). We observed that miR-155 was the only miRNA predicted to target the *CTLA4* 3’UTR by all three prediction tools. To test the involvement of miRNAs in melanoma secretome-mediated *CTLA4* mRNA instability, we cultured human Treg cells in the absence or presence of 50% 1205Lu-MCM for 24 h and analyzed mature miRNA expression by RT-PCR. We found that miR-155 was highly expressed in untreated Treg cells and was further upregulated in Treg cells treated with MCM was highly expressed among these putative miRNAs in untreated Treg cells, whereas other putative miRNAs were downregulated or upregulated to some extent without statistical significance in Treg cells treated with MCM (**Figure 6C–I**). The complementary alignment of the miR-155 seed sequence with the human *CTLA4* 3’UTR sequence with a 7mer site (**Supplementary Figure 5**) supports that miR-155 targets the 3’UTR of *CTLA4* mRNA. These data suggest that melanoma cell secretome affects blood *CTLA4* expression through miRNA-mediated gene-silencing, specifically miR-155.

### 3.6 Upregulation of miR-155 in treg cells of metastatic melanoma patients results in *CTLA4* degradation

**Figure 7:**
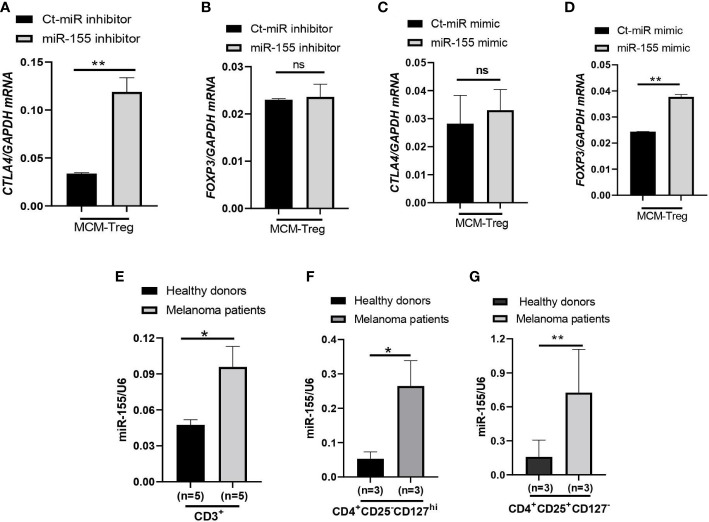
miR-155 regulation of *CTLA4* mRNA stability in human Treg cells cultured in MCM and its expression in immune cells of metastatic melanoma patients. **(A, B)** qRT-PCR analysis of *CTLA4* **(A)** and *FOXP3* **(B)** expression in human Treg cells transfected with 50 nM control (Ct-MiR) or miR-155 inhibitors and subsequently cultured in 50% control media + 50% 1205Lu-MCM (MCM-Treg) for 24 h. **(C, D)** qRT-PCR analysis of *CTLA4* **(C)** and *FOXP3* **(D)** expression in human Treg cells transfected with 50 nM control or miR-155 mimics and subsequently cultured in 50% control media + 50% 1205Lu-MCM (MCM-Treg) for 24 h. *CTLA4* and *FOXP3* mRNA levels were normalized with internal control *GAPDH* expression. **(E–G)** Expression of miR-155 in auto-MACS-fractionated CD3^+^ T cells **(E)**, CD4^+^ T cells **(F)**, and CD4^+^CD25^+^ T cells **(G)** from healthy donors (n = 3) and metastatic melanoma patients (n = 5 and 3 in E and F-G, respectively). The expression of miR-155 was normalized using U6 snRNA as an internal control. Representative data are shown and expressed as the mean ± SEM (n = 3). ns, not significant, *p < 0.05, and ** *p* < 0.01.

To investigate the role of miR-155 in regulating *CTLA4* mRNA expression, we transfected human Treg cells with miR-155 inhibitors or miR-155 mimics and cultured them in the presence of 50% 1205Lu-MCM for 24 h. Gene expression analysis of *CTLA4* and *FOXP3* in Treg cells showed that transfection of human Treg cells with miR-155 inhibitor rescued downregulated *CTLA4* expression without affecting the expression of *FOXP3* (**Figures 7A, B**), supporting the idea that miR-155 targets *CTLA4* but not *FOXP3*. On the other hand, transfection of human Treg cells with miR-155 mimics in the presence of 50% 1205Lu-MCM did not affect *CTLA4* expression (**Figure 7C**) but significantly upregulated *FOXP3* expression (**Figure 7D**), suggesting that the effect of miR-155 on *CTLA4* is limited when the gene expression is already downregulated or miR-155 requires additional factors to be effective. To elucidate the former possibility, we transfected miR-155 mimics into control Treg cells that did not have downregulated *CTLA4*. The transfection of miR-155 mimics did not significantly affect the expression of *CTLA4* in these control Treg cells (**Supplementary Figure 6**). These findings suggest that miR-155 plays a critical role in downregulating *CTLA4* mRNA in MCM-treated Treg cells but additional factors may be necessary for miR-155 to downregulate *CTLA4* in Treg cells from melanoma.

Furthermore, we analyzed miR-155 expression levels in peripheral CD3^+^ cells, CD4^+^ cells, and CD4^+^ CD25^+^ cells from melanoma patients and compared them with healthy donors. The results showed that miR-155 expression was upregulated in these T cells in melanoma patients (**Figures 7E–G**).

## 4 Discussion

In the current study, we investigated the levels of *CTLA4* mRNA expression and found that low levels of *CTLA4* expression in both tumor tissue and blood cells are associated with poorer overall survival in patients with metastatic melanoma. The study also uncovered that reduced *CTLA4* expression in the blood cells of these patients is due to Treg cells, whose *CTLA4* was silenced at the post-transcriptional level by miR-155. Functionally, we demonstrated that *CTLA4* expression inhibits the proliferation and suppressive function of human Treg cells. These findings shed new light on the pathophysiology of metastatic melanoma and could potentially inform the development of innovative immune-based therapies.

We observed a significant decrease in the *CTLA4* levels in Treg cells from melanoma patients, which were almost equivalent to the levels observed in Tconv cells. CTLA-4 is a major immune checkpoint expressed in activated T cells and constitutively in Treg cells (7), but its role in Treg cells has been debated. Germline deletion of *Ctla4* resulted in severe autoimmunity with lethality (17, 18), which prompted researchers to delineate the effects of *Ctla4* on T cells and Treg cells separately. Tang et al. showed normal development, homeostasis, and uncompromised suppressive activity in *Ctla4*-deficient Treg cells from germline-depleted mice (37). Similarly, Schmidt et al. showed an increased peripheral Treg cell population in germline-depleted mice; however, this phenotype was not observed in thymic Treg cells (38). These data suggest that CTLA-4 negatively regulates the peripheral Treg cells’ expansion, and that downregulated CTLA-4 may drive the proliferation of circulating Treg cells without decreasing their suppressive function. The conditional ablation of *Ctla4* in adult Treg cells protected mice from autoimmunity (20), supporting the notion that CTLA-4 is a negative regulator of Treg cell expansion. Our data demonstrated that *CTLA4* expression inhibits the proliferation and suppressive function of human Treg cells, suggesting that CTLA-4 is a negative regulator of Treg cell function.

**Figure 8:**
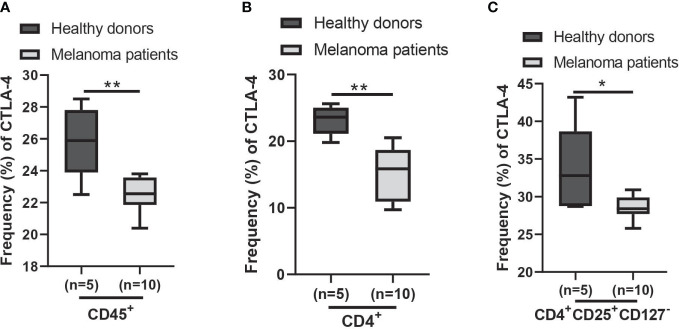
Expression of CTLA-4 protein in blood samples of healthy donors and metastatic melanoma patients. Analysis of blood CTLA-4 expression in immune cell subsets of metastatic melanoma patients using the publicly available CyTOF data (dataset #2 results of (32)). The percent frequency of CTLA-4-expressing cells in CD45^+^ cells **(A)**, CD4^+^ T cells **(B)**, and CD4^+^CD25^+^CD127^-^ T cells (Treg cells) **(C)** from healthy donors (n = 5) and metastatic melanoma patients (n = 10). Blood samples were obtained before the immunotherapy. Representative data are shown and expressed as the mean ± SEM, *p < 0.05 and ** *p* < 0.01.

Using the AUS cohort, we observed a decrease in *CTLA4* expression in the blood of melanoma patients, and this reduction was associated with a poorer prognosis for patients with metastatic melanoma. Similar results were observed from the US cohort, but they were not supported by the statistical analysis. Further analysis of cohort characteristics indicated that the US cohort was skewed slightly younger and had higher levels of log *CTLA4* compared to the AUS cohort (data not shown), which might have led to marginal disconcordance between two cohorts. Furthermore, the data from the AUS cohort indicate that blood *CTLA4* levels determine the melanoma patient prognosis independent of age and sex, specifically in the AUS cohort. To confirm these results, we analyzed a publicly available CyTOF dataset (32) and found that CTLA-4 protein levels were reduced in circulating immune cells (CD45^+^), CD4^+^ T cells, and Treg cells (CD4^+^CD25^+^CD127^-^) in metastatic melanoma patients compared to healthy donors (**Figures 8A–C**). The same paper also reported that higher CTLA-4 protein levels in peripheral CD4^+^ and CD8^+^ T cells were associated with a better response to anti-PD1 therapy in metastatic melanoma (32). Additionally, another study reported that *CTLA4* methylation in tumors, which leads to reduced *CTLA4* mRNA, is associated with resistance to anti-PD-1 and anti-CTLA-4 immunotherapy in melanoma patients (15). Therefore, the lack of upregulation of CTLA-4 in either peripheral blood or tumors might make inhibition of not only this molecule but also other checkpoints such as PD-1 ineffective and decrease their efficacy as monotherapy. The mechanism of reducing *CTLA4* mRNA expression in either peripheral blood or tumors may be related to resistance to immunotherapy.

Here, we provide mechanistic insights into the downregulation of *CTLA4* expression in Treg cells from melanoma through miR-155. Various mechanisms upregulate *CTLA4* expression. TCR triggering and CD28 co-stimulation upregulate *CTLA4* in T cells (39), and tumor cell-intrinsic *CTLA4* upregulation can be induced by β-catenin signaling (35). However, Treg cells constitutively express *CTLA4* due to the transcription factor FOXP3 (19). In contrast to the upregulation of *CTLA4*, downregulation of *CTLA4* expression can occur not only due to epigenetic changes, such as promoter methylation (15), but also through RNA interference, which affects the stability of expressed *CTLA4* mRNA, as seen in our current study. Recent studies have highlighted the role of miRNAs in regulating immune checkpoint (PD-1, PD-L1, and CTLA-4) gene expression and their importance as regulators of T-cells and tumor cells (40). While some miRNAs, such as miRs-9, -105, -155, and -487a-3p, directly modulate *CTLA4* expression (40, 41), others like miRs-24 and -210 indirectly downregulate *CTLA4* through direct downregulation of FOXP3 (42). Moreover, miR-155 has been shown to directly target the 3’UTR of *CTLA4* in CD8^+^CD25^+^ Treg cells (43) and helper T-cells, increasing their proliferative response by downregulation of *CTLA4* (44). Consistent with this, our study confirmed that miR-155 downregulates *CTLA4* at the mRNA level without interfering with *FOXP3* expression in circulating Treg cells from melanoma patients. miRNAs regulate target genes through the miRNA-induced silencing complex (miRSC), which interacts with various molecules, such as RNA-binding proteins, poly (A)-binding proteins, and GW182 (45). While miR-155 inhibitor rescued downregulated *CTLA4* in Treg cells treated with MCM, miR-155 mimic did not affect *CTLA4* expression even in control naïve Treg cells. These data suggest that miR-155 is one of the major factors regulating the stability of blood *CTLA4* mRNA in melanoma but additional factors may be necessary for miR-155 to play this critical role. Further identification of co-factors that regulate *CTLA4* mRNA stability in Treg cells from melanoma patients is needed to fully understand the mechanisms involved.

Furthermore, we show that melanoma secretome induces downregulation of *CTLA4* through miR-155 expression in Treg cells without decreasing their *FOXP3* expression. Similar to other miRNAs, the function of miR-155 is context-dependent (46). While miR-155 has been reported to suppress tumors by dampening anti-tumor immunity in T cells (47), it has also been shown to suppress T helper cell activation (48), enhance myeloid-derived suppressor cells (49), and expand Treg cells (50), indicating its oncogenic role. The induction of miR-155 is mediated by various signaling pathways, including transforming growth factor (TGF) β through Smad4 (51). TGFβ is a major component of the secretome from melanoma cells (25), and has been shown to induce miR-155 expression in various immune cells. Therefore, TGFβ in melanoma secretome may induce miR-155 intrinsically in Treg cells, leading to downregulating their *CTLA4* expression. This could explain why we observed higher upregulation of miR-155 in the T cells and Treg cells from melanoma patients than in healthy donors. Moreover, miR-155 is transcriptionally induced by FOXP3 and is expressed more highly in Treg cells than in other CD4 T cells (52). miR-155 promotes suppressive competence and proliferation by inhibiting the suppressors of cytokine signaling (SOCS)1 in Treg cells (53). Similar to our study, *CTLA4* is also shown to be a direct target of miR-155 (43). These studies suggest that downregulated *CTLA4 via* miR-155 predominantly contributes to the expansion of Treg cells without affecting their suppressive function. In support of this, Paterson and colleagues showed that Treg cells with conditional ablation of *Ctla4* in adult mice remained functionally suppressive and sufficient to protect mice from experimental autoimmune encephalomyelitis (20). CTLA-4 is a negative regulator of Treg cell homeostasis and their cell proliferation (54), and anti-CTLA-4 treatment in tumor models enhances the Treg cell population even though it yields tumor-specific immune responses (55), opposing their therapeutic outcomes in tumor studies. Our study reveals that the post-transcriptional silencing of *CTLA4* by miRNA-155 in Treg cells may contribute to reducing *CTLA4* mRNA expression observed in melanoma patients. These findings suggest that targeting miRNA-155 or other factors involved in regulating *CTLA4* expression in Treg cells without affecting T cells could be a potential approach for improving the efficacy of immunotherapy in melanoma.

## 5 Conclusions

Our study provides new insights into the underlying mechanisms of reduced *CTLA4* expression observed in melanoma patients, demonstrating that post-transcriptional silencing of *CTLA4* by miR-155 in Treg cells may play a critical role. Our findings suggest that targeting miR-155 or other factors involved in regulating *CTLA4* expression in Treg cells, without affecting T cells, could be a potential strategy to improve the efficacy of immunotherapy in melanoma. These results may have broader implications for other cancers where immune checkpoint inhibition has been shown to be less effective, as these phenotypes have also been observed in anti-PD-1 therapy. Further research is needed to understand the molecular mechanisms that regulate *CTLA4* and miR-155 expression in Treg cells and identify potential therapeutic targets for enhancing immune-based therapies.

## Data availability statement

The original contributions presented in the study are included in the article/**Supplementary Material**. Further inquiries can be directed to the corresponding author/s.

## Ethics statement

The studies involving human participants were reviewed and approved by Human Research Ethics Committee of Edith Cowan University (No. 2932) and Sir Charles Gardner Hospital (No. 2007-123) for AUS cohort. The US cohort was approved by the Institutional Review Boards of the University of Colorado (COMIRB#05-0309). Blood from healthy donors was collected at the Children’s Hospital Blood Donor Centre in Aurora, CO, USA, approved under COMIRB#17–0110. The patients/participants provided their written informed consent to participate in this study.

## Author contributions

Conceptualization, MF. Methodology, PV, DO, AN, DS. Formal analysis, PV, AN, DM, DG, ZZ. Data collection, DS, JM, AR, JD, GN, WR, MZ. Data curation, PV, AN, DG, ZZ, MF. Writing—original draft preparation, PV, AN, NW, JM, ZZ. Writing—review and editing, PV, DO, DM., DG, ZZ, MF. Supervision, MF. Funding acquisition, MZ, MF. All authors contributed to the article and approved the submitted version.

## References

[1] Whiteman DC Green AC Olsen CM. The growing burden of invasive melanoma: projections of incidence rates and numbers of new cases in six susceptible populations through 2031. J Invest Dermatol (2016) 136(6):1161–71. doi: 10.1016/j.jid.2016.01.035 26902923

[2] O’Neill CH Scoggins CR. Melanoma. J Surg Oncol (2019) 120(5):873–81. doi: 10.1002/jso.25604 31246291

[3] Siegel RL Miller KD Wagle NS Jemal A. Cancer statistics, 2023. CA Cancer J Clin (2023) 73(1):17–48. doi: 10.3322/caac.21763 36633525

[4] Carlino MS Larkin J Long GV. Immune checkpoint inhibitors in melanoma. Lancet (2021) 398(10304):1002–14. doi: 10.1016/S0140-6736(21)01206-X 34509219

[5] Lenschow DJ Walunas TL Bluestone JA. CD28/B7 system of T cell costimulation. Annu Rev Immunol (1996) 14:233–58. doi: 10.1146/annurev.immunol.14.1.233 8717514

[6] Leach DR Krummel MF Allison JP. Enhancement of antitumor immunity by CTLA-4 blockade. Science (1996) 271(5256):1734–6. doi: 10.1126/science.271.5256.1734 8596936

[7] Chambers CA Kuhns MS Egen JG Allison JP. CTLA-4-mediated inhibition in regulation of T cell responses: mechanisms and manipulation in tumor immunotherapy. Annu Rev Immunol (2001) 19:565–94. doi: 10.1146/annurev.immunol.19.1.565 11244047

[8] Hodi FS O’Day SJ McDermott DF Weber RW Sosman JA Haanen JB. Improved survival with ipilimumab in patients with metastatic melanoma. N Engl J Med (2010) 363(8):711–23. doi: 10.1056/NEJMoa1003466 PMC3549297 20525992

[9] Lipson EJ Drake CG. Ipilimumab: an anti-CTLA-4 antibody for metastatic melanoma. Clin Cancer Res (2011) 17(22):6958–62. doi: 10.1158/1078-0432.CCR-11-1595 PMC3575079 21900389

[10] Weber J Mandala M Del Vecchio M Gogas HJ Arance AM Cowey CL. Adjuvant nivolumab versus ipilimumab in resected stage III or IV melanoma. N Engl J Med (2017) 377(19):1824–35. doi: 10.1056/NEJMoa1709030 28891423

[11] Larkin J Chiarion-Sileni V Gonzalez R Grob JJ Rutkowski P Lao CD. Five-year survival with combined nivolumab and ipilimumab in advanced melanoma. N Engl J Med (2019) 381(16):1535–46. doi: 10.1056/NEJMoa1910836 31562797

[12] Liu Y Zheng P. Preserving the CTLA-4 checkpoint for safer and more effective cancer immunotherapy. Trends Pharmacol Sci (2020) 41(1):4–12. doi: 10.1016/j.tips.2019.11.003 31836191 PMC7210725

[13] Maibach F Sadozai H Jafari SMS Hunger RE Schenk M. Tumor-infiltrating lymphocytes and their prognostic value in cutaneous melanoma. Front Immunol (2020) 11. doi: 10.3389/fimmu.2020.02105 PMC7511547 33013886

[14] Van Allen EM Miao D Schilling B Shukla SA Blank C Zimmer L. Genomic correlates of response to CTLA-4 blockade in metastatic melanoma. Science (2015) 350(6257):207–11. doi: 10.1126/science.aad0095 PMC5054517 26359337

[15] Goltz D Gevensleben H Vogt TJ Dietrich J Golletz C Bootz F. CTLA4 methylation predicts response to anti-PD-1 and anti-CTLA-4 immunotherapy in melanoma patients. JCI Insight (2018) 3(13):e96793. doi: 10.1172/jci.insight.96793 PMC6124533 29997292

[16] Fietz S Zarbl R Niebel D Posch C Brossart P Gielen GH. CTLA4 promoter methylation predicts response and progression-free survival in stage IV melanoma treated with anti-CTLA-4 immunotherapy (ipilimumab). Cancer Immunol Immun (2021) 70(6):1781–8. doi: 10.1007/s00262-020-02777-4 PMC8139923 33196890

[17] Waterhouse P Penninger JM Timms E Wakeham A Shahinian A Lee KP. Lymphoproliferative disorders with early lethality in mice deficient in ctla-4. Science (1995) 270(5238):985–8. doi: 10.1126/science.270.5238.985 7481803

[18] Tivol EA Borriello F Schweitzer AN Lynch WP Bluestone JA Sharpe AH. Loss of ctla-4 leads to massive lymphoproliferation and fatal multiorgan tissue destruction, revealing a critical negative regulatory role of ctla-4. Immunity (1995) 3(5):541–7. doi: 10.1016/1074-7613(95)90125-6 7584144

[19] Wing K Onishi Y Prieto-Martin P Yamaguchi T Miyara M Fehervari Z. CTLA-4 control over Foxp3(+) regulatory T cell function. Science (2008) 322(5899):271–5. doi: 10.1126/science.1160062 18845758

[20] Paterson AM Lovitch SB Sage PT Juneja VR Lee Y Trombley JD. Deletion of CTLA-4 on regulatory T cells during adulthood leads to resistance to autoimmunity. J Exp Med (2015) 212(10):1603–21. doi: 10.1084/jem.20141030 PMC4577848 26371185

[21] Cancer Genome Atlas N. Genomic classification of cutaneous melanoma. Cell (2015) 161(7):1681–96. doi: 10.1016/j.cell.2015.05.044 PMC4580370 26091043

[22] Cerami E Gao J Dogrusoz U Gross BE Sumer SO Aksoy BA. The cBio cancer genomics portal: an open platform for exploring multidimensional cancer genomics data. Cancer Discov (2012) 2(5):401–4. doi: 10.1158/2159-8290.CD-12-0095 PMC3956037 22588877

[23] Cirenajwis H Ekedahl H Lauss M Harbst K Carneiro A Enoksson J. Molecular stratification of metastatic melanoma using gene expression profiling: prediction of survival outcome and benefit from molecular targeted therapy. Oncotarget (2015) 6(14):12297–309. doi: 10.18632/oncotarget.3655 PMC4494939 25909218

[24] Luo YC Robinson S Fujita J Siconolfi L Magidson J Edwards CK. Transcriptome profiling of whole blood cells identifies PLEK2 and C1QB in human melanoma. PloS One (2011) 6(6):e20971. doi: 10.1371/journal.pone.0020971 21698244 PMC3115966

[25] Osborne DG Domenico J Luo Y Reid AL Amato C Zhai Z. Interleukin-37 is highly expressed in regulatory T cells of melanoma patients and enhanced by melanoma cell secretome. Mol Carcinog. (2019) 58(9):1670–9. doi: 10.1002/mc.23044 PMC6692223 31099111

[26] Okamoto M Liu W Luo Y Tanaka A Cai X Norris DA. Constitutively active inflammasome in human melanoma cells mediating autoinflammation *via* caspase-1 processing and secretion of interleukin-1beta. J Biol Chem (2010) 285(9):6477–88. doi: 10.1074/jbc.M109.064907 PMC2825443 20038581

[27] McGeary SE Lin KS Shi CY Pham TM Bisaria N Kelley GM. The biochemical basis of microRNA targeting efficacy. Science (2019) 366(6472):eaav1741. doi: 10.1126/science.aav1741 PMC7051167 31806698

[28] John B Enright AJ Aravin A Tuschl T Sander C Marks DS. Human microRNA targets (vol 2, pg 1862, 2005). PloS Biol (2005) 3(7):1328. doi: 10.1371/journal.pbio.0020363 PMC521178 15502875

[29] Chen K Rajewsky N. Natural selection on human microRNA binding sites inferred from SNP data. Nat Genet (2006) 38(12):1452–6. doi: 10.1038/ng1910 17072316

[30] Feng B Cao Y Chen S Chu X Chu Y Chakrabarti S. miR-200b mediates endothelial-to-Mesenchymal transition in diabetic cardiomyopathy. Diabetes (2016) 65(3):768–79. doi: 10.2337/db15-1033 26718496

[31] Zhai ZL Samson JM Yamauchi T Vaddi PK Matsumoto Y Dinarello CA. Inflammasome sensor NLRP1 confers acquired drug resistance to temozolomide in human melanoma. Cancers (2020) 12(9):2518. doi: 10.3390/cancers12092518 PMC7563249 32899791

[32] Krieg C Nowicka M Guglietta S Schindler S Hartmann FJ Weber LM. High-dimensional single-cell analysis predicts response to anti-PD-1 immunotherapy. Nat Med (2018) 24(2):144–53. doi: 10.1038/nm.4466 29309059

[33] Spidlen J Brinkman RR. Use FlowRepository to share your clinical data upon study publication. Cytom Part B-Clin Cy. (2018) 94(1):196–8. doi: 10.1002/cyto.b.21393 27342384

[34] Olsen CM Thompson JF Pandeya N Whiteman DC. Evaluation of sex-specific incidence of melanoma. JAMA Dermatol (2020) 156(5):553–60. doi: 10.1001/jamadermatol.2020.0470 PMC7097866 32211827

[35] Spranger S Bao R Gajewski TF. Melanoma-intrinsic beta-catenin signalling prevents anti-tumour immunity. Nature (2015) 523(7559):231–5. doi: 10.1038/nature14404 25970248

[36] Li XJ Wang XY Cheng ZN Zhu QB. AGO2 and its partners: a silencing complex, a chromatin modulator, and new features. Crit Rev Biochem Mol (2020) 55(1):33–53. doi: 10.1080/10409238.2020.1738331 32164444

[37] Tang Q Boden EK Henriksen KJ Bour-Jordan H Bi M Bluestone JA. Distinct roles of CTLA-4 and TGF-beta in CD4+CD25+ regulatory T cell function. Eur J Immunol (2004) 34(11):2996–3005. doi: 10.1002/eji.200425143 15468055

[38] Schmidt EM Wang CJ Ryan GA Clough LE Qureshi OS Goodall M. CTLA-4 controls regulatory T cell peripheral homeostasis and is required for suppression of pancreatic islet autoimmunity. J Immunol (2009) 182(1):274–82. doi: 10.4049/jimmunol.182.1.274 19109158

[39] Carreno BM Collins M. The B7 family of ligands and its receptors: new pathways for costimulation and inhibition of immune responses. Annu Rev Immunol (2002) 20:29–53. doi: 10.1146/annurev.immunol.20.091101.091806 11861596

[40] Kipkeeva F Muzaffarova T Korotaeva A Mansorunov D Apanovich P Nikulin M. The features of immune checkpoint gene regulation by microRNA in cancer. Int J Mol Sci (2022) 23(16):9324. doi: 10.3390/ijms23169324 36012588 PMC9409052

[41] Skafi N Fayyad-Kazan M Badran B. Immunomodulatory role for MicroRNAs: regulation of PD-1/PD-L1 and CTLA-4 immune checkpoints expression. Gene (2020) 754:144888. doi: 10.1016/j.gene.2020.144888 32544493

[42] Fayyad-Kazan H Rouas R Fayyad-Kazan M Badran R El Zein N Lewalle P. MicroRNA profile of circulating CD4-positive regulatory T cells in human adults and impact of differentially expressed microRNAs on expression of two genes essential to their function. J Biol Chem (2012) 287(13):9910–22. doi: 10.1074/jbc.M111.337154 PMC3323050 22294691

[43] Jebbawi F Fayyad-Kazan H Merimi M Lewalle P Verougstraete JC Leo O. A microRNA profile of human CD8(+) regulatory T cells and characterization of the effects of microRNAs on treg cell-associated genes. J Transl Med (2014) 12:1–6. doi: 10.1186/s12967-014-0218-x PMC4440568 25090912

[44] Sonkoly E Janson P Majuri ML Savinko T Fyhrquist N Eidsmo L. MiR-155 is overexpressed in patients with atopic dermatitis and modulates T-cell proliferative responses by targeting cytotoxic T lymphocyte-associated antigen 4. J Allergy Clin Immun (2010) 126(3):581–U306. doi: 10.1016/j.jaci.2010.05.045 20673989

[45] Duchaine TF Fabian MR. Mechanistic insights into MicroRNA-mediated gene silencing. Csh Perspect Biol (2019) 11(3):a032771. doi: 10.1101/cshperspect.a032771 PMC6396329 29959194

[46] Svoronos AA Engelman DM Slack FJ. OncomiR or tumor suppressor? the duplicity of MicroRNAs in cancer. Cancer Res (2016) 76(13):3666–70. doi: 10.1158/0008-5472.CAN-16-0359 PMC4930690 27325641

[47] Huffaker TB Lee SH Tang WW Wallace JA Alexander M Runtsch MC. Antitumor immunity is defective in T cell-specific microRNA-155-deficient mice and is rescued by immune checkpoint blockade. J Biol Chem (2017) 292(45):18530–41. doi: 10.1074/jbc.M117.808121 PMC5682963 28912267

[48] Goncalves-Alves E Saferding V Schliehe C Benson R Kurowska-Stolarska M Brunner JS. MicroRNA-155 controls T helper cell activation during viral infection. Front Immunol (2019) 10:1367. doi: 10.3389/fimmu.2019.01367 31275315 PMC6593301

[49] Chen SQ Wang L Fan J Ye C Dominguez D Zhang Y. Host miR155 promotes tumor growth through a myeloid-derived suppressor cell-dependent mechanism. Cancer Res (2015) 75(3):519–31. doi: 10.1158/0008-5472.CAN-14-2331 PMC4315710 25502838

[50] Schjenken JE Moldenhauer LM Zhang BH Care AS Groome HM Chan HY. MicroRNA miR-155 is required for expansion of regulatory T cells to mediate robust pregnancy tolerance in mice. Mucosal Immunol (2020) 13(4):609–25. doi: 10.1038/s41385-020-0255-0 31988469

[51] Kong W Yang H He L Zhao JJ Coppola D Dalton WS. MicroRNA-155 is regulated by the transforming growth factor beta/Smad pathway and contributes to epithelial cell plasticity by targeting RhoA. Mol Cell Biol (2008) 28(22):6773–84. doi: 10.1128/MCB.00941-08 PMC2573297 18794355

[52] Kohlhaas S Garden OA Scudamore C Turner M Okkenhaug K Vigorito E. Cutting edge: the Foxp3 target miR-155 contributes to the development of regulatory T cells. J Immunol (2009) 182(5):2578–82. doi: 10.4049/jimmunol.0803162 19234151

[53] Lu LF Thai TH Calado DP Chaudhry A Kubo M Tanaka K. Foxp3-dependent microRNA155 confers competitive fitness to regulatory T cells by targeting SOCS1 protein. Immunity (2009) 30(1):80–91. doi: 10.1016/j.immuni.2008.11.010 19144316 PMC2654249

[54] Tang AL Teijaro JR Njau MN Chandran SS Azimzadeh A Nadler SG. CTLA4 expression is an indicator and regulator of steady-state CD4(+)FoxP3(+) T cell homeostasis. J Immunol (2008) 181(3):1806–13. doi: 10.4049/jimmunol.181.3.1806 PMC2683757 18641318

[55] Marangoni F Zhakyp A Corsini M Geels SN Carrizosa E Thelen M. Expansion of tumor-associated treg cells upon disruption of a CTLA-4-dependent feedback loop. Cell (2021) 184(15):3998. doi: 10.1016/j.cell.2021.05.027 34157302 PMC8664158

