# Supplementary material for: CTLA4 mRNA is downregulated by miR-155 in regulatory T cells, and reduced blood CTLA4 levels are associated with poor prognosis in metastatic melanoma patients
Source: Front Immunol. 2023 May 1;14:1173035. doi: 10.3389/fimmu.2023.1173035 (PMC10183574; doi:10.3389/fimmu.2023.1173035)
Supplement: Supplementary file 1 [file DataSheet_1.docx]

Supplementary Material

*CTLA4* mRNA Is Downregulated by miR-155 in Regulatory T Cells, and Reduced Blood *CTLA4* Levels Are Associated With Poor Prognosis in Metastatic Melanoma Patients

**Prasanna Kumar Vaddi ^1^, Douglas Grant Osborne ^1^, Andrew Nicklawsky ^2^, Nazanin Kalani Williams ^1^, Dinoop Ravindran Menon ^1^, Derek Smith ^1^, Jonathan Mayer ^1^, Anna Reid ^3^, Joanne Domenico ^1^, Giang Huong Nguyen ^1^, William A Robinson ^3^, Melanie Ziman ^4, 5^, Dexiang Gao ^2^, Zili Zhai ^1^, Mayumi Fujita ^1, 6, 7 *^**

*** Correspondence:** Mayumi Fujita
[mayumi.fujita@cuanschutz.edu](mailto:mayumi.fujita@cuanschutz.edu)

# Supplementary Figures and Tables

## Supplementary Figures


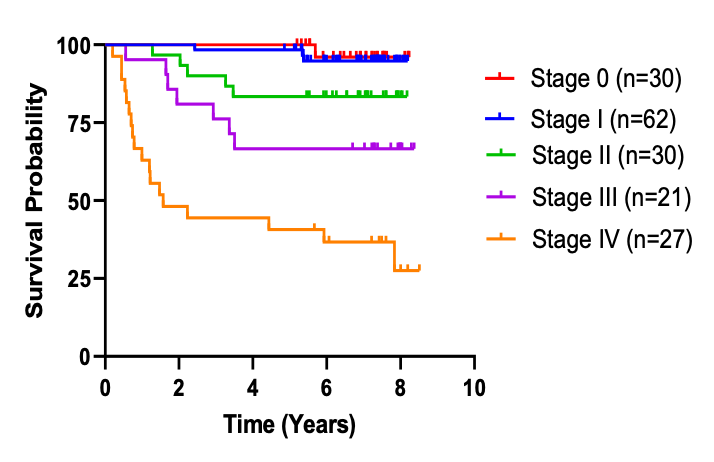


**Figure S1:** K-M survival plot of the AUS cohort based on the melanoma stage.

**Figure S2: The time-course of MCM-mediated *CTLA4* expression in human Treg cells.** qRT-PCR analysis of *CTLA4* expression in human Treg cells cultured in 100% lymphocyte culture media (Ct-Treg) or 50% MCM + 50% lymphocyte culture media (MCM-Treg) at various time points (0–96 h). The expression of *CTLA4* was normalized using *GAPDH* as an internal control. Representative data are shown and expressed as the mean ± SEM, ns: not significant, **p* < 0.05, and ** *p* < 0.01.


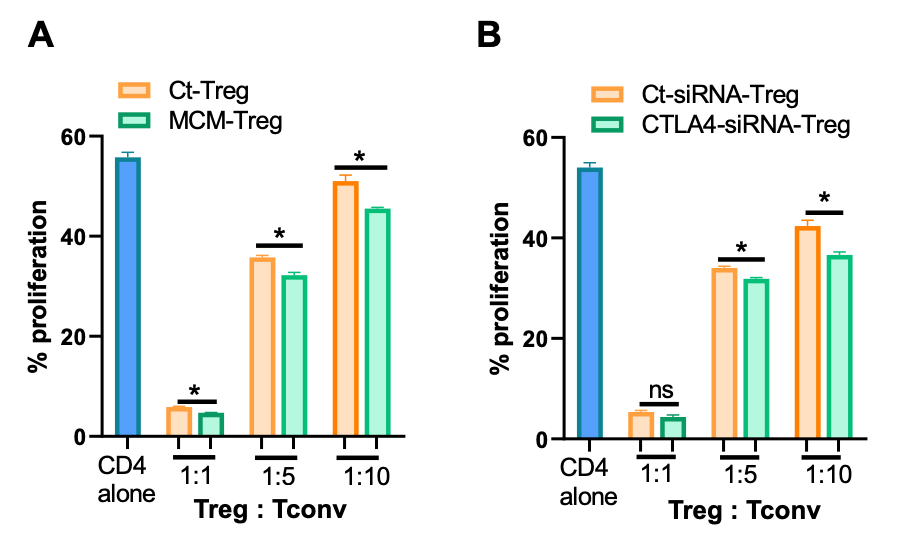


**Figure S3:** **Proliferation index of CFSE-labeled human Tconv cells co-cultured with human Treg cells.** (**A**) The bar graph representation of the proliferation index of CFSE-labeled Tconv cells co-cultured with control Tregs cells (orange) or MCM-treated Treg cells (green) at different ratios (1:1, 1:5 and 1:10, Treg:Tconv) or CFSE-labeled Tconv cells alone (blue) in lymphocyte culture media with CD3/28 beads and rhIL-2 for 72 h. (**B**) The bar graph representation of the proliferation index of CFSE-labeled Tconv cells co-cultured with control-siRNA transfected Tregs cells (orange) or *CTLA4*-siRNA transfected Treg cells (green) at different ratios (1:1, 1:5 and 1:10, Treg:Tconv) or CFSE-labeled Tconv cells alone (blue) in lymphocyte culture media with CD3/28 beads and rhIL-2 for 72 h. Representative data are shown and expressed as the mean ± SEM (n = 3). ns: not significant, and **p* < 0.05.

**Figure S4:** **Knockdown of *CTLA4* in human Treg cells.** qRT-PCR analysis of *CTLA4* expression from human Treg cells transfected with control siRNA or *CTLA4* siRNA and cultured for 24 h. The expression of *CTLA4* was normalized using *GAPDH* as an internal control. Representative data are shown and expressed as the mean ± SEM, *** *p* < 0.01.


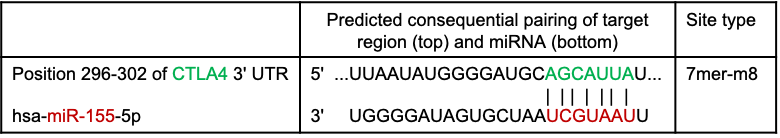


**Figure S5**: **Complimentary alignment of miR-155 with sequence from the *CTLA4* 3' UTR.** Alignment of miR-155 with sequence from the *CTLA4* mRNA 3' UTR, predicted by TargetScan bioinformatic tool.

**Figure S6:** qRT-PCR analysis of *CTLA4* expression from human Treg cells transfected with control miR-mimic (Ct-miR-mimic) or miR-155-mimic and cultured in lymphocyte culture media for 24 h. The expression of *CTLA4* was normalized using *GAPDH* as an internal control. Representative data are shown and expressed as the mean ± SEM, ns: not significant.

## Supplementary Tables

**Table S1.** Primers used for qRT-PCR.

| Gene | Forward | Reverse |
| --- | --- | --- |
| *CTLA4* | CTCAGCTGAACCTGGCTACC | CTTCAGTCACCTGGCTGTCA |
| *GAPDH* | TGCACCACCAACTGCTTAGC | GGCATGGACTGTGGTCATGAG |
| *AGO2* | CCACCTAGACCCGACTTTGG | CCACCTTGTCCCTCCCAATC |
| *FOXP3* | TCGAAGAGCCAGAGGACTTC | GATGATGCCACAGATGAAGC |

**Table S2**. Overview *CTLA4* for AUS and US patients stratified with melanoma status.

|  |  | **AUS Cohort** | |
| --- | --- | --- | --- |
|  |  | Non-melanoma | Melanoma |
|  |  | N = 103 | N = 170 |
| *CTLA4* (median [IQR]) |  | 0.01  [0.00, 0.01] | 0.00  [0.00, 0.01] |
| Log *CTLA4* (median [IQR]) |  | -5.24  [-5.58, -4.92] | -5.63  [-6.00, -5.29] |
| Sex (%) | Female | 63 (61.2) | 66 (38.8) |
|  | Male | 40 (38.8) | 104 (61.2) |
| Age (median [IQR]) |  | 45.00  [32.00, 61.50] | 66.00  [57.00, 78.00] |
